# Preparation of Granular Organic Iodine and Selenium Complex Fertilizer Based on Biochar for Biofortification of Parsley

**DOI:** 10.1155/2024/6601899

**Published:** 2024-10-21

**Authors:** Yerlan Doszhanov, Meiram Atamanov, Jakpar Jandosov, Karina Saurykova, Zhandos Bassygarayev, Adilkhan Orazbayev, Seitzhan Turganbay, Aitugan Sabitov

**Affiliations:** ^1^Nanobiotechnology Laboratory, Institute of Combustion Problems, Almaty 050012, Kazakhstan; ^2^The Faculty of Chemistry and Chemical Technology, Al-Farabi Kazakh National University, Almaty 050040, Kazakhstan; ^3^Department of Chemistry, Kazakh National Women's Teacher Training University, Almaty 050000, Kazakhstan; ^4^Department of General University Studies, Egyptian University of Islamic Culture Nur-Mubarak, Almaty 050040, Kazakhstan; ^5^Kazakh-British Technical University, “One Belt, One Road” Petroleum Engineering, Almaty 050000, Kazakhstan

**Keywords:** agricultural enhancement, antioxidant properties, biochar, iodine-enriched biochar, parsley growth, selenium-enriched biochar

## Abstract

This study investigates the potential of biochar derived from various biomass sources: apricot kernel (AK), pine sawdust (PS), rice husk (RH), wheat straw (WS), and reed stem (RS) to enhance the yield, nutritional quality, and environmental sustainability of parsley crops. Comprehensive characterization through SEM, EDAX, nitrogen adsorption–desorption isotherms, and FTIR analyses identified AK biochar as the most suitable for further enrichment due to its superior specific surface area (512.3 m^2^/g) and iodine number (51.23 mg/g). EDAX analysis revealed that AK biochar exhibited the highest carbon content (92.1%), while RH biochar contained the highest silicon content (46%), indicating different potential applications. FTIR analysis identified key functional groups, such as carbonyl (1740 cm⁻^1^) and hydroxyl (3430 cm⁻^1^) groups, which contribute to the biochar's reactivity and potential effectiveness in various applications. The effects of selenium (Na_2_SeO_4_), iodine (KI), their combination (Na_2_SeO_4_ + KI), and BISF (biochar-enriched iodine and selenium fertilizer) on parsley growth, antioxidant properties, and nutrient accumulation were evaluated. The results demonstrated that joint applications of iodide and selenate led to a 3.1-fold increase in iodine content (up to 16.8 mg/kg d.w.) and a 1.2-fold increase in selenium accumulation (up to 2482.1 *μ*g/kg d.w.) in parsley compared to separate treatments. Additionally, BISF treatment significantly improved key biometric parameters, with leaf weight increasing by 1.6 times (up to 326.5 g) compared to the control, and antioxidant content—ascorbic acid, polyphenols, and antioxidant activity—showing increases of 1.56, 1.27, and 1.50 times, respectively. This study underscores the effectiveness of selenium- and iodine-enriched biochar in enhancing parsley crop yield and nutritional quality while also demonstrating the multifunctional role of biochar in environmental remediation.

## 1. Introduction

For the Republic of Kazakhstan, the problem of iodine deficiency is relevant for agriculture and has a high social importance. A study conducted by the Kazakh Academy of Nutrition found that in most parts of Kazakhstan, there is a lack of iodine in local food products (owing to low levels of the microelement in soil and water, respectively). The foci of endemic goiter were registered in 11 of 14 regions, and the intensity of their spread throughout the regions increased to the south and east of the country. According to the results of the study, Kazakhstan was classified as a country with a moderate endemicity of iodine deficiency diseases [1, 2]. While iodized salt has been widely implemented as a public health measure to address iodine deficiency, relying solely on this source may not sufficiently meet the daily iodine requirements for the general population [3]. Several factors contribute to this limitation. Firstly, the growing trend toward reduced salt intake due to concerns over hypertension and cardiovascular diseases has led to lower overall iodine consumption. Secondly, the iodine content in salt can vary significantly, and iodine losses during cooking and storage can further reduce its efficacy. Additionally, certain populations, such as those with dietary restrictions, thyroid conditions, or who consume minimally processed foods, may not receive adequate iodine from iodized salt alone [4]. These challenges highlight the need for alternative iodine sources, such as iodine-enriched foods, to ensure adequate iodine intake.

Unlike potassium, nitrogen, or phosphorus, for plants, iodine and selenium are not the main elements necessary to increase productivity. However, selenium has been established to increase the antioxidant protection of plants against drought, salinity, high light or ultraviolet radiation, characteristic of the climate, and soil of the Republic of Kazakhstan [5–7]. Research from several authors has confirmed that presowing treatment of seeds of various crops with a solution of potassium iodide significantly enriches food products with iodine; sugar beets on medium-podzolic loam under the influence of potassium iodide increased the yield of roots and increased their sugar content. Seaweed containing iodine also increased the yield of the roots of this root vegetable. Potatoes in light soddy-podzolic soils under the influence of iodine increased tuber yield and increased their starchy content and potassium content. Corn, under the influence of potassium iodide foliar feeding, increases grain yield [8–10]. Furthermore, in small concentrations, iodine and selenium compounds cause a biostimulating effect in agricultural crops and increase the content of antioxidants in the plant, growth stimulants, flavonoids, and hormones, ensuring the productivity and nutritional value of agriculture [11–15].

Fertilizers based on potassium iodide or potassium iodate are now widely studied and used. There is also research and development on the use of organic salts as fertilizers, for example, 3,5-diiodo-4-hydroxybenzonitrile (ioxynil). However, the above-mentioned inorganic and organic compounds of iodine, also as selenium salts, are highly soluble in water and, without carriers, are subject to rapid leaching from the soil, and as a result, they must be used in higher concentrations than that is necessary for plant physiology.

The process of leaching of iodine and selenium can be controlled through the use of sorbents. The ability to design the surface of sorbents will make it possible to control the release of components and simulate the process of action of microelements. Different crops mature at different times, and micronutrient treatment is required at different times. The use of biochar-based fertilizers will allow us to simulate the release time of iodine and the selenium microelement in the required time periods [16–18].

In this work, the effectiveness of the use of biochar-based granular iodine–selenium complex fertilizer (BISF) on the biochemical characteristics of plants and the level of accumulation of trace elements of iodine and selenium by plants was investigated. The object of the study was curly parsley (*Petroselinum crispum*), a 1- to 2-year-old plant of the *Umbelliferae* family, a popular culinary seasoning with a high content of antioxidants: furocoumarin bergapten and flavone glycoside apiin [19].

## 2. Materials and Methods

### 2.1. Materials

Characteristics of biochars obtained from the pyrolysis of biomass from the next plant residues: rice husk (RH) of the genus *Oryza sativa*, reed stem (RS) of the genus *Phragmites australis*, pine sawdust (PS) of the genus *Pinus sylvéstri*, wheat straw (WS) of the genus *Trticum aestvum*, and apricot kernels (AK) of the genus *Prunus armeniaca.*

### 2.2. Obtaining Biochar From Plant Residues

The preparation of biochar samples from plant biomass is carried out in two stages. 5 g of conditioned (precleaned and selected) plant waste is mixed with powdered KOH in a biomass/alkali ratio of 1:1, 1:2, 1:3, and 1:4 by weight. Next, it is preheated to 80°C for 3 h to ensure the access of KOH to the biomass. A weighed amount of the impregnated material is then placed in a vertical electric oven with air supply.

In the second stage, the pyrolysis of the samples is carried out at temperatures from 300°C to 850°C with a heating rate of 5°C/min. After reaching maximum temperature, the samples are kept for 3 h and then cooled to room temperature. The cooled material is thoroughly washed with a 0.1 M hydrochloric acid solution and distilled water to remove alkali residues. At the same time, the pH value of the washed solution is monitored. The optimal pH value is 6–7. After the washing process, the biochar samples were dried at a temperature of 100°C–105°C.

### 2.3. Study of the Physicochemical and Technological Properties of Biochars

The chemical composition of the samples was investigated by scanning electron microscopy (SEM) “QUANTA 3D 200i” device (FEI, USA), coupled with EDAX (energy-dispersive X-ray analysis) spectrometer and a semiconductor-based detector with an energy tolerance of 128 eV (polymer material, sensitive area *d* = 0.3 mm) [20].

Infrared spectra of the samples were recorded using a Spectrum 65, Perkin&Elmer 1100 FTIR (Fourier-transform infrared) spectrometer over the range of 4000 − 400 cm⁻^1^, employing KBr pellets with a resolution of 1 cm⁻^1^. The pellet for infrared studies was prepared by mixing a given sample with KBr crystals and pressed into a pellet.

The bulk density of biochar samples (kg/m^3^) was determined by weighing a measuring cylinder filled with a substance according to the following formula [21]:

(1) $$\begin{array}{c} \rho_{b}=\frac{m}{V}, \end{array}$$

where *ρ*_*b*_ is the bulk density of the biochar sample, kg/m^3^; *m* is the mass of biochar, kg; and *V* is the volume of biochar in the cylinder after presealing, m^3^.

The granulometric composition of biochar was determined using vibrational sieve analysis on the VP-30T device (Russia). A sample of the material is placed on the upper sieve of the device; then, the device is switched on, and the entire set of sieves vibrates for 30 min. The remaining samples in each sieve are weighed with electronic scales, and the yield of each class in grams and as a percentage of the total mass of the sample is recorded in the table [22].

The porosity of the biochar was determined by BET-sorptometer. The nitrogen adsorption–desorption process was carried out at a temperature of −196°C using a specific surface analyzer “Sorptometer-M” [23, 24].

### 2.4. Study of the Sorption Capacity of Biocarbons in Relation to Iodine

The iodine number is a widely used method for determining the adsorption capacity because of its simplicity and rapid assessment of the quality of the activated carbonized sorbent. The method is based on the study of a three-point adsorption isotherm. The biochar attachments are impregnated with an iodine solution at room temperature and the resulting mixtures are filtered. The number of iodines in the filtrate is determined by titration and is expressed in milligrams per 1 g of biochar or in % (weight fractions) at an iodine concentration of 0.02 mol/L. The concentration of standard iodine solution should be monitored and should be (0.100 + 0.001) mol/L for all analyses [25].

### 2.5. Production of Mineral Complex Organic Fertilizer Under Laboratory Conditions

The closest in terms of technical essence of the mineral complex fertilizer that we are developing is a method for producing granular fertilizer based on biochar containing the trace element iodine, as KI [26]. We modified this method, polyvinyl alcohol was used as a hydrophilizing agent instead of silica, and an organic iodide complex was added di-(2S)-2-amino-3-(1H-indole-3-yl)propionate))-dihydro-tetraiodide with antiviral and antimicrobial properties to protect plants from diseases [27, 28].

Previously, biochar was obtained from the AK. In 100 mL of distilled water 14 g of di-((2S)-2-amino-3-(1H-indole-3-yl) propionate)-dihydro-tetraiodide, 20 g of potassium iodide; 0.3 g of polyvinyl alcohol; 0.1 g of sodium selenate were dissolved. Biochar was added to the resulting ready-made solution in a ratio of 1:1 by weight, mixed, and dried with hot air until a crumbly mass was formed with a humidity of 17%–20%. Next, the granulation of the resulting dried material was carried out in a cone granulator until granules with diameter and length of up to 5–6 mm were obtained.

### 2.6. Planting of Plants

Curly parsley (*Petroselinum crispum*) was grown under laboratory conditions in plastic pots with drainage holes at the bottom, with a volume of 2 L (diameter 10 cm) on a mixture of turf and zeolite. Soil characteristics where Curly parsley seeds planted were: humus content up to 15%; soil humidity—no more than 35%; pH—5.0–6.0; 12.2 mg/kg mineral nitrogen; 20.2 mg/kg ammonium ions; 365 mg/kg phosphorous compounds; 198 mg/kg potassium ions. For additional illumination, ULI-P20-18W/SPSB IP40 white fluorescent lamps were installed at a height of 30 cm from the upper leaves, with a total maximum power of 35 W.

The experiment included the biofortification of parsley with iodine and selenium without and against the background of BISF treatment.

The experiment included six options: (1) control; (2) Na_2_SeO_4_ at a concentration of 10 mg/L; (3) KI at a concentration of 100 mg/L; (4) KI + Na_2_SeO_4_; (5) BISF with a total iodine content of 10% and selenium of 1%; and (6) pure biochar.

### 2.7. Sample Preparation of Plant Specimens

After harvesting, the petioles of the plants were washed with distilled water to remove soil residues, and the leaves and petioles were separated, weighed, and homogenized. Fresh homogenates were used to determine the content of ascorbic acid and photosynthetic pigments.

The rest of the material was dried at 50°C to a constant weight to further determine the content of nitrates, water-soluble compounds, antioxidant activity (AOA), and polyphenols.

The dry matter content was determined by gravimetric method after drying the samples at 50°C to a constant mass [29].

### 2.8. Determination of Ascorbic Acid Concentration

The concentration of ascorbic acid was determined by a titrimetric method using Tilmans reagent (2,6-sodium dichlorophenolindophenol) [30].

### 2.9. Determination of the Content of Polyphenols in Plant Samples

Polyphenol concentration in plant samples was determined using a spectrophotometer using Folin–Ciocalteu reagent [31]. 1 g of the dried parsley sample was extracted for 1 h at 70°C with 20 mL of 70% ethyl alcohol solution. The extract was cooled to 22°C–25°C, quantitatively transferred to a 50-mL volumetric flask, and brought to the mark with 70% alcohol. The final solution was mixed and filtered through a folded filter. 1 mL of the final extract, 2.5 mL of saturated sodium carbonate solution, and 0.25 mL of Folin–Ciocalteu reagent diluted 1:1 with distilled water were added to a 25-mL volumetric flask. The resulting reaction mixture was stirred and brought to the mark with distilled water. 1 h after the end of the reaction, the absorption index was measured at 730 nm on a SF-56 spectrophotometer (OKB Spektr, Russia). The polyphenol content was calculated using a standard curve obtained using 6 standard solutions of gallic acid in the concentration range of 0–90 *μ*g/mL. The results of the determination were expressed in mg-eq of gallic acid/g dry weight (mg GA/g d.r.).

### 2.10. Determination of the AOA of Samples

To determine the AOA of the samples, a colorimetric method [32] was used based on titration of a 0.01 N potassium permanganate solution in an acid medium with ethanol parsley extract before discoloration, indicating a complete reduction of Mn^+7^ to Mn^+2^. Gallic acid was used as the standard of comparison. The results of the determination of AOA were expressed in milligrams of gallic acid/g of dry weight (mg GA/g of dr).

### 2.11. Determination of the Total Iodine Content

Total iodine was determined using an Ecotest voltammetric analyzer (Russia) [33] equipped with a three-electrode electrochemical cell: auxiliary and reference electrodes (silver chlorides in 1 M KCl), as well as a silver electrode. 2 mL of 10% potassium hydroxide solution was added to 0.1 g of the homogenized dried sample, and the resulting mixture was mineralized at 550°C. The reaction mass was cooled, and 1 mL of 10% zinc sulfate solution was added. The resulting sample was dissolved in 10 mL of distilled water, and the iodine concentration was determined using formic acid as a background electrolyte and standard solutions of potassium iodide 0.1 mg/L, 1 mg/L, and 10 mg/L.

### 2.12. Statistical Analysis

Statistical differences between measurements were determined by analysis of variance (ANOVA) using Origin (data analysis software). Average values were compared using the least significant difference (LSD) test at *p* < 0.05.

## 3. Results and Discussions

### 3.1. Investigation of the Physicochemical Characteristics of Biochar Samples

Pyrolyzed plant parts were selected as natural plant sources: RH, PS, RS, AK, and WS. The main physicochemical characteristics of different samples of biochar are presented in Table 1.

The bulk density for carbonized samples of RH, PS, AK, RS, and WS was 0.423 kg/m^3^, 0.482 kg/m^3^, 0.517 kg/m^3^, 0.396 kg/m^3^, and 0.388 kg/m^3^, respectively. The biochar of RH consisted of fine particles with a size of 0.80 mm. The particle size of the sorbent from the PS of the pine and AKs was commensurate, and the average size was in the range of 1.8–2.5 mm. Carbonized WS, such as the stem of the reed, was easily crushed and contained more dust, and the average particle size was 0.35–0.5 mm.

Biocarbon samples from PS, RH, and AK are characterized by a developed specific surface area and in this indicator exceed other comparison samples by at least 2–3 times. The porous structure of biochars is mainly represented by micropores, which account for 70.2%–81.0% of the total volume of micropores.

### 3.2. Comprehensive Characterization of Biochar: Structural, Elemental, Adsorption, and Functional Group Analysis

**Figure 1:**
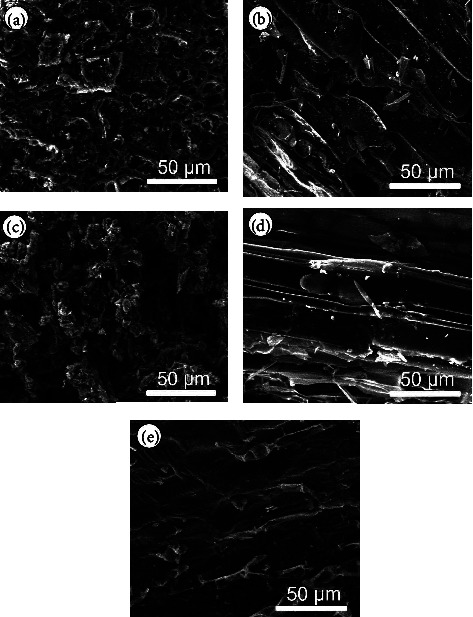
SEM image of the biochar structure (scale 1:10,000) of (a) AK, (b) PS, (c) RH, (d) WS, and (e) RS. AK; apricot kernels of the genus *Prunus armeniaca*, PS; pine sawdust of the genus *Pinussylv'estri*, RH; rice husk of the genus *Oryza sativa*, RS; reed stem of the genus *Phragmites australis*, WS; wheat straw of the genus *Trticum aestvum*.

Figure 1 presents SEM images of biochar samples derived from various plant materials. Figure 1(a) shows that the AK biochar exhibits a highly porous structure with numerous small cavities and interconnected networks. This high degree of porosity is likely due to the thermal decomposition of organic matter during pyrolysis, which results in a lightweight and high-surface-area material. Such a structure is beneficial for applications requiring high adsorption capacity, such as pollutant removal from water or air.

Figure 1(b) displays the PS biochar, which shows a more fibrous and less porous structure compared to AK biochar. The fibrous nature of this biochar may be attributed to the preservation of the original cellular structure of the wood during pyrolysis. This structure can enhance the mechanical strength of the biochar, making it suitable for use in soil amendment, where structural integrity is important for maintaining soil aeration and water retention.

Figure 1(c) illustrates the RH biochar, which displays a rough and granular surface morphology with a high degree of fragmentation. The granular structure may result from the high silica content in RH, which contributes to the formation of rigid, brittle particles during pyrolysis. This structure is advantageous for applications where the biochar needs to provide a high surface area for interaction with pollutants or nutrients.

Figure 1(d) shows the WS biochar, which has a more elongated and layered structure, with visible striations and channels along the surface. This morphology suggests that the original fibrous structure of the WS was partially preserved during pyrolysis. The layered structure may provide channels for water and nutrient movement, making this biochar particularly useful in agricultural applications.

Figure 1(e) depicts the RS biochar, which also shows a fibrous structure, but with more visible cracks and fractures compared to PS biochar. These features suggest that the pyrolysis process caused some degree of structural collapse, leading to the formation of cracks. Despite this, the fibrous structure remains intact, indicating that this biochar could still offer benefits in terms of soil aeration and water retention [34, 35].

**Figure 2:**
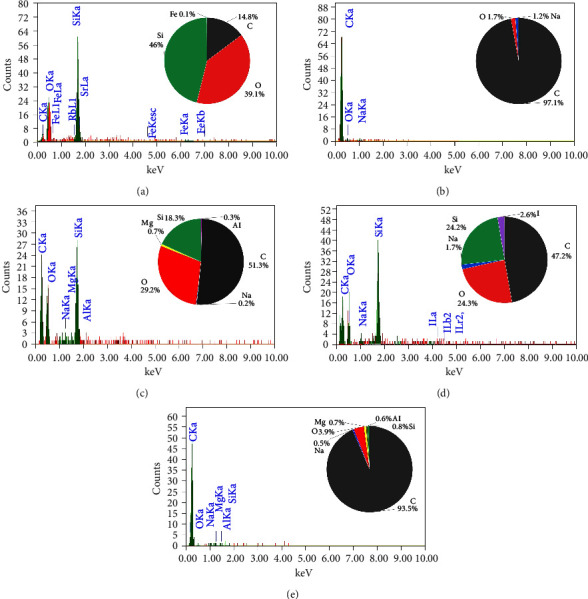
EDAX spectra and elemental composition of biochars derived from RH (a), AK (b), RS (c), WS (d), and PS (e). AK; apricot kernels of the genus *Prunus armeniaca*, PS; pine sawdust of the genus *Pinussylv'estri,* RH; rice husk of the genus *Oryza sativa*, RS; reed stem of the genus *Phragmites australis*, WS; wheat straw of the genus *Trticum aestvum*.

The EDAX spectra presented in Figure 2 provide a detailed comparison of the elemental composition of biochar samples derived from various plant materials. Each spectrum is accompanied by a pie chart that visually represents the relative abundance of elements detected in each sample, offering insights into the compositional differences that arise from the use of different feedstocks and pyrolysis conditions.

The elemental composition of the RH biochar (Figure 2(a)) reveals a significant presence of silicon (46%), oxygen (14.8%), and carbon (39.1%), with a small amount of iron (0.1%). The high silicon content is characteristic of RH, which is known for its high silica (SiO_2_) content. In contrast, the AK biochar (Figure 2(b)) is overwhelmingly composed of carbon (92.1%), with minor contributions from oxygen (1.7%) and sodium (1.2%). The high carbon content reflects the efficient carbonization process during pyrolysis. The low oxygen content suggests a minimal presence of oxidized compounds, which may enhance the biochar's stability and resistance to degradation.

The RS biochar (Figure 2(c)) shows a balanced composition with significant amounts of carbon (51.7%), oxygen (29.3%), and silicon (15.9%), along with trace amounts of magnesium (0.7%) and aluminum (0.3%). This biochar's composition indicates a mix of organic and inorganic components, with silica being a notable constituent. WS biochar (Figure 2(d)) exhibits a composition with carbon (47.2%) and silicon (24.2%) as major components, alongside oxygen (24.3%), sodium (1.7%), and iodine (2.6%). The high silicon content, similar to that of RH biochar, suggests potential use in enhancing soil structure. The presence of iodine is particularly interesting, as it may have been introduced during the biochar production process or through contamination. Finally, the PS biochar (Figure 2(e)) is predominantly composed of carbon (93.5%), with oxygen (5.6%), silicon (0.5%), magnesium (0.3%), and aluminum (0.1%) making up the remainder. The high carbon content indicates that PS biochar, like AK biochar, is highly carbon-rich and stable, making it suitable for applications focused on carbon sequestration.

RH and WS biochars are rich in silicon, which may be beneficial for specific soil enhancement applications [36, 37]. In contrast, AK and PS biochars are highly carbon-rich, making them ideal for carbon sequestration. RS biochar offers a balanced composition, potentially serving as both a carbon source and a provider of essential minerals.

**Figure 3:**
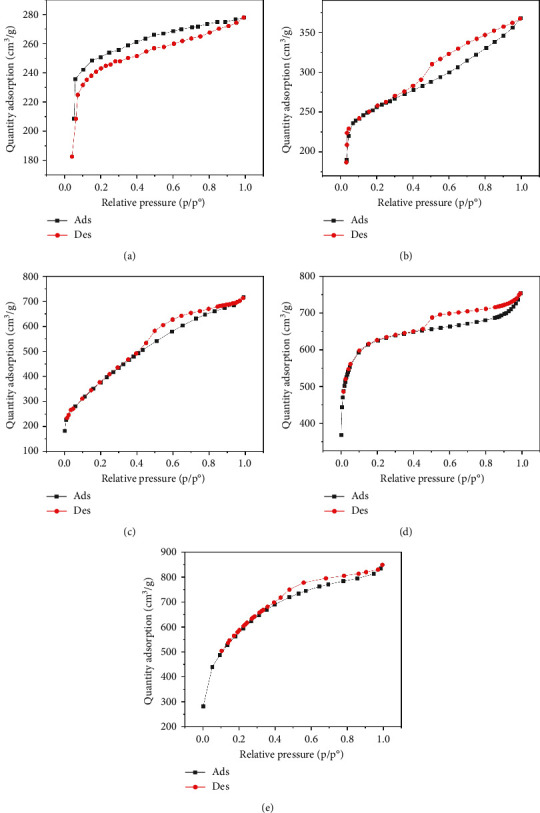
Nitrogen adsorption–desorption isotherms of biochars derived from wheat straw (a), RS (b), RH (c), PS (d), and AK (e). AK; apricot kernels of the genus *Prunus armeniaca*, PS; pine sawdust of the genus *Pinussylv'estri*, RH; rice husk of the genus *Oryza sativa*, RS; reed stem of the genus *Phragmites australis*, WS; wheat straw of the genus *Trticum aestvum*.

Figure 3 presents the nitrogen adsorption–desorption isotherms of five different biochar samples derived from various plant materials: WS (a), RS (b), RH (c), PS (d), and AK (e). These isotherms are crucial for characterizing the porosity and surface area of the biochar samples, which directly influence their effectiveness in applications such as adsorption, soil amendment, and carbon sequestration.

The isotherms display characteristic Type IV shapes, which are indicative of mesoporous materials. This suggests that all five biochar samples possess a significant amount of mesopores, contributing to their adsorption capacity. The hysteresis loops observed between the adsorption and desorption branches in each plot further confirm the presence of mesoporous structures, with differences in the shape and size of the loops indicating variations in pore structure among the samples.

For WS biochar (Figure 3(a)), the isotherm shows a gradual increase in adsorption at lower relative pressures, followed by a more rapid uptake as the pressure increases, indicating a wide distribution of pore sizes. RS biochar (Figure 3(b)) exhibits a similar trend, although the adsorption capacity appears slightly lower, suggesting smaller or fewer pores compared to WS biochar.

RH biochar (Figure 3(c)) and PS biochar (Figure 3(d)) both demonstrate higher adsorption capacities, with isotherms extending to larger quantities of adsorbed nitrogen. This indicates a greater surface area and/or pore volume, making these biochars particularly effective for applications that require high adsorption capacity. The isotherm for AK biochar (Figure 3(e)) shows the highest overall adsorption, suggesting an even larger surface area and possibly more extensive pore development, which is consistent with its high carbon content observed in the EDAX analysis.

These adsorption–desorption characteristics align with the elemental compositions discussed earlier, where biochars with higher carbon content, such as those derived from AK and PS, exhibited greater adsorption capacities. This suggests that the effectiveness of these biochars in applications such as pollutant removal or soil amendment could be closely linked to their porosity and surface chemistry, both of which are influenced by the type of biomass and pyrolysis conditions used during their production.

**Figure 4:**
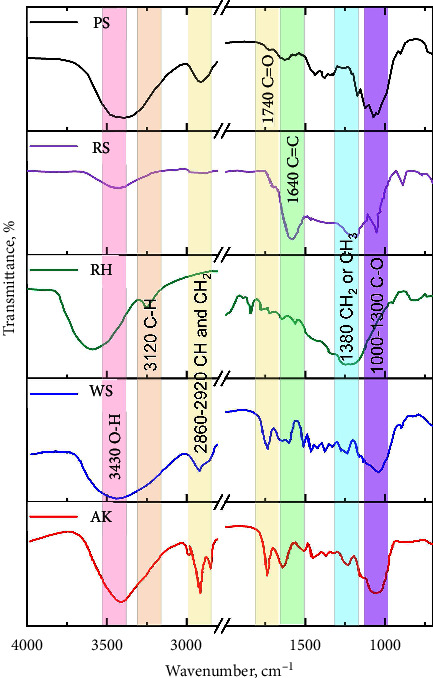
FTIR spectra of biochars derived from WS, RS, RH, PS, and AK. AK; apricot kernels of the genus *Prunus armeniaca*, PS; pine sawdust of the genus *Pinussylv'estri*, RH; rice husk of the genus *Oryza sativa*, RS; reed stem of the genus *Phragmites australis*, WS; wheat straw of the genus *Trticum aestvum*.

Figure 4 presents the FTIR spectra of biochar samples derived from five different plant materials: WS, RS, RH, PS, and AK. The spectrum for PS shows a significant absorption band around 1740 cm⁻^1^, which is attributed to the C=O stretching vibrations of carbonyl groups [38]. This suggests the presence of carboxylic acids, esters, or aldehydes, which could be remnants of the original biomass or products of the pyrolysis process. The spectrum for RS shows a prominent absorption band at 1640 cm⁻^1^, corresponding to C=C stretching vibrations, typically associated with alkenes or aromatic rings [39]. This indicates the presence of unsaturated carbon structures, which could result from the breakdown of lignin or other aromatic compounds in the biomass. The FTIR spectrum of RH biochar displays bands around 3120 cm⁻^1^ and 2860–2920 cm⁻^1^, corresponding to C-H stretching vibrations in alkanes, and 1380 cm⁻^1^, which can be attributed to CH_3_ or CH_2_ groups.

These features indicate the presence of aliphatic hydrocarbons, which are likely derived from the cellulose and hemicellulose components of the RH. WS biochar exhibits a broad absorption band at 3430 cm⁻^1^, characteristic of O-H stretching vibrations, which indicates the presence of hydroxyl groups. This band is likely associated with residual moisture or the presence of phenolic compounds. Additionally, the band at 2860–2920 cm⁻^1^ corresponds to C-H stretching, further indicating the presence of aliphatic hydrocarbons [40]. The FTIR spectrum of AK biochar shows various peaks, including those around 1740 cm⁻^1^ (C=O stretching) and 1380 cm⁻^1^ (CH_3_ or CH_2_ groups), indicating the presence of carbonyl compounds and aliphatic hydrocarbons [41]. The spectrum suggests a complex mixture of functional groups, likely due to the high carbon content and diverse chemical composition of the AK.

These FTIR spectra highlight the diverse chemical compositions of the biochar samples, which are influenced by the nature of the original biomass and the conditions under which pyrolysis occurred. The presence of specific functional groups such as carbonyls, hydroxyls, and aliphatic hydrocarbons provides valuable information about the reactivity and potential applications of these biochars in environmental and agricultural contexts.

The differences observed in the FTIR spectra of these biochars can also be correlated with their elemental compositions and adsorption properties, as discussed earlier. For instance, biochars with prominent C=O and O-H groups may exhibit higher reactivity in soil amendment applications, while those with a higher degree of aromaticity may offer enhanced stability and carbon sequestration potential.

### 3.3. Investigation of the Sorption Capacity of Biochar Samples in Relation to Iodine

One of the ultimate goals of this study is the development of a mineral complex and organic fertilizer, which is planned to be obtained in the form of a granular powder. The basis of the granular powder will be biochar obtained from plant waste, which should be characterized by developed porosity, large specific surface area, and high sorption activity in relation to the sorbed substances. The iodine number is a widely used method for determining the sorption capacity because of its simplicity and rapid assessment of the quality of biochar.

**Figure 5:**
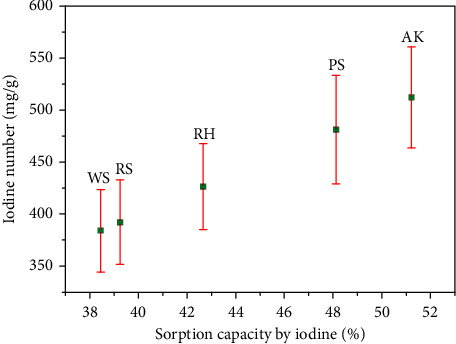
Correlation between sorption capacity by iodine and iodine number for various biochar samples.

The correlation between the sorption capacity by iodine and the iodine number for various biochar samples is depicted in Figure 5. The biochar types investigated include RH, RS, PS, WS, and AK. Error bars are included to indicate the variability in iodine number measurements, reflecting the precision of the experimental data.

The data reveal a positive correlation between the sorption capacity by iodine (expressed as a percentage) and the iodine number (in mg/g). Specifically, biochars with higher sorption capacities generally exhibit higher iodine numbers, suggesting that these biochars possess more developed porosity and larger specific surface areas, which enhance their iodine adsorption efficiency.

Among the samples, the AK biochar demonstrates the highest performance, with a sorption capacity of 51.23% and an iodine number of 512.3 mg/g. This indicates that AK biochar has the most developed pore structure, making it highly effective in iodine adsorption. The distinct separation of error bars for the AK sample compared to others suggests that its superior sorption performance is statistically significant.

PS biochar also shows a relatively high iodine number of 481.3 mg/g and a sorption capacity of 48.13%, indicating good adsorption properties. In contrast, WS biochar exhibits the lowest sorption capacity (38.44%) and iodine number (384.4 mg/g) among the samples, suggesting less favorable structural properties for iodine adsorption.

RH and RS biochars display moderate sorption capacities and iodine numbers, with values of 42.66% and 426.6 mg/g, and 39.25% and 392.5 mg/g, respectively. The similarity in their adsorption characteristics suggests comparable structural features, which may be attributed to the raw material composition or production processes.

The error bars indicate some overlap in the iodine number measurements, particularly between the RS and WS biochars, implying that the differences in their adsorption efficiencies may not be statistically significant. However, the higher performance of the AK biochar is clearly distinguished from the others.

### 3.4. Effect of Selenium, Iodine, and BISF on the Biometric Parameters of Parsley and Dry Matter Content

**Figure 6:**
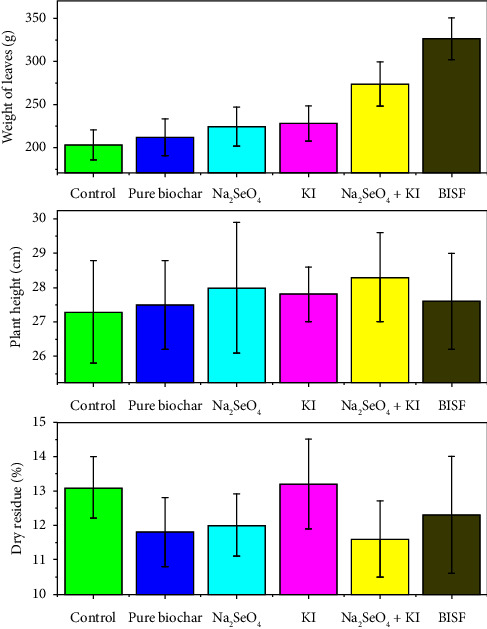
Effects of different treatments by selenium (Na_2_SeO_4_), iodine (KI), their combination (Na_2_SeO_4_ + KI), and BISF on three key biometric parameters of parsley.

The bar charts presented in Figure 6 illustrate the effects of different treatments—selenium (Na_2_SeO_4_), iodine (KI), their combination (Na_2_SeO_4_ + KI), and BISF—on three key biometric parameters of parsley: weight of leaves, plant height, and dry residue content. The weight of parsley leaves is a critical indicator of the plant's overall growth and biomass production. The data reveal that the application of BISF resulted in the highest leaf weight (326.5 g), representing a significant increase compared to the control (203.0 g). The combination treatment of Na_2_SeO_4_ + KI also enhanced leaf weight substantially (273.8 g), suggesting that the synergistic effects of selenium and iodine can boost plant growth. In contrast, the individual treatments (pure biochar, Na_2_SeO_4_, and KI) showed only moderate increases in leaf weight, indicating that while these treatments positively impact growth, they are less effective than the combined or BISF treatments.

Plant height is another important metric of vegetative growth, and in this study, the results show that the treatments had a relatively modest impact on this parameter. Across all treatments, including the control, plant height remained fairly consistent, ranging from 27.3 cm to 28.3 cm. This suggests that while selenium and iodine treatments, particularly in the form of BISF, significantly increase leaf biomass, they do not necessarily lead to taller plants. This outcome might be attributed to the specific action of these treatments in enhancing leaf growth rather than elongation.

Dry residue percentage is a measure of the plant's dry matter content, which reflects its structural composition and potential nutritional value. The results indicate that the treatments had a varied effect on dry residue content. The control and KI treatment showed the highest dry residue percentages (13.1% and 13.2%, respectively), while the BISF treatment resulted in a slightly lower dry residue content (12.3%). Interestingly, the Na_2_SeO_4_ + KI treatment led to the lowest dry residue percentage (11.6%), suggesting a trade-off between increased leaf biomass and dry matter concentration. This could imply that while these treatments enhance growth, they may lead to a more succulent plant with a higher water content.

The bar charts provide a clear comparative view of how different treatments affect the growth and composition of parsley. The BISF treatment consistently outperforms the other treatments in terms of increasing leaf biomass, making it the most effective treatment overall. However, the relatively stable plant height across treatments suggests that the primary impact of these treatments is on leaf growth rather than overall plant size.

The Na_2_SeO_4_ + KI combination also shows strong performance in increasing leaf weight, though it appears to decrease the dry matter content. This could be an important consideration for agricultural practices, depending on whether the goal is to maximize fresh weight or dry matter content.

The relatively modest effects of the individual treatments (pure biochar, Na_2_SeO_4_, and KI) on these biometric parameters highlight the importance of using integrated approaches like BISF for achieving more significant improvements in plant growth and yield.

The findings suggest that the application of BISF or the combined treatment of Na_2_SeO_4_ + KI can significantly enhance parsley biomass, making these treatments valuable for agricultural practices aimed at increasing yield. However, the choice of treatment should consider the specific agricultural goals, such as whether the priority is fresh weight, dry matter content, or a balance of both. The potential trade-offs observed, such as the decrease in dry residue content with increased biomass, should be carefully managed to align with the desired outcomes in crop production.

### 3.5. Comparative Analysis of Antioxidant Properties in Parsley Treated With Selenium, Iodine, and BISF

**Figure 7:**
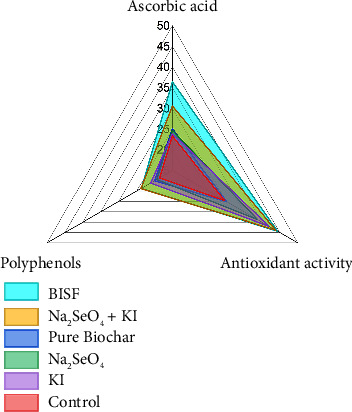
Comparative analysis of antioxidant properties in parsley treated with selenium, iodine, and BISF.

The possibility of increasing the level of natural antioxidants in plants when treated with selenium and iodine is widely discussed in the scientific literature [42]. The radar plot (Figure 7) provides a comprehensive comparison of the antioxidant properties in parsley when treated with various combinations of selenium, iodine, and BISF. The three key antioxidant parameters analyzed include ascorbic acid content, AOA, and polyphenol content.

Ascorbic acid, commonly known as vitamin C, plays a crucial role in plant defense mechanisms and human nutrition. The radar plot reveals that the BISF treatment resulted in the highest ascorbic acid content (36.4 mg/100 g d.w.), significantly surpassing all other treatments. The combination of Na_2_SeO_4_ + KI also led to a substantial increase in ascorbic acid content (30.6 mg/100 g d.w.), indicating a synergistic effect when selenium and iodine are applied together. In contrast, the control and other individual treatments (pure biochar, Na_2_SeO_4_, and KI) resulted in comparatively lower ascorbic acid levels, underscoring the enhanced efficacy of combined treatments.

AOA, measured in mg GA/g d.w., reflects the overall ability of the plant to neutralize free radicals. The radar plot shows that both the Na_2_SeO_4_ + KI and BISF treatments significantly enhanced the AOA of parsley, with values reaching 43.9 and 44.4 mg GA/g d.w., respectively. These results suggest that the combined application of selenium and iodine, particularly in the BISF formulation, is highly effective in boosting the antioxidant capacity of parsley. The individual application of KI also resulted in a notable increase in AOA (42.0 mg GA/g d.w.), although slightly less than the combined treatments.

Polyphenols are important secondary metabolites that contribute to the antioxidant defense system of plants and provide numerous health benefits. The radar plot indicates that the Na_2_SeO_4_ + KI and BISF treatments led to the highest polyphenol content, with values of 23.8 and 23.7 mg GA/g d.w., respectively. The control group exhibited the lowest polyphenol content (18.6 mg GA/g d.w.), suggesting that the treatments significantly improved the polyphenolic profile of parsley.

The radar plot clearly illustrates the superior performance of the BISF treatment across all three antioxidant parameters. This treatment not only maximizes ascorbic acid content but also enhances the AOA and polyphenol levels, making it the most effective among the tested options. The combination of Na_2_SeO_4_ + KI also shows a strong overall profile, indicating that the synergistic effects of selenium and iodine contribute significantly to the antioxidant properties of parsley. The control and individual treatments (pure biochar, Na_2_SeO_4_, and KI) exhibit lower antioxidant properties than the combined treatments, reinforcing the importance of using integrated approaches like BISF for maximizing the nutritional and health-promoting properties of parsley.

The findings from this analysis suggest that the use of BISF and combined selenium–iodine treatments can substantially enhance the antioxidant properties of parsley. This has important implications for both agricultural practices and human nutrition, as these treatments could be employed to produce parsley with higher health benefits. The enhanced antioxidant profile achieved through these treatments could also contribute to the plant's resilience against environmental stresses, further supporting their use in sustainable agricultural practices. In this work, a significant increase in the content of ascorbic acid was detected in parsley leaves when treating plants with potassium iodide + sodium selenate, as well as when treating BISF: 1.31 and 1.56 times, respectively. All treatment options for the joint application of iodide with selenate led to an increase in iodine levels in plants compared to the separate application of iodide (Table 2).

The data obtained indicate the effective interaction of trace elements (I and Se) on the level of enrichment of plants with iodine both when administered as a solution and when administered as part of BISF. Thus, during the treatment of iodide + selenate, the iodine content increased by 2.3 times and in the case of administration as part of BISF by 3.1 compared to the treatment with separate iodine. Considering that the dry mass yield of parsley is 13%, as well as the recommended daily human need for iodine is 120 mcg and selenium is 50 mcg, 25 g of parsley enriched with iodide + selenite can provide up to 55% of the daily human need for iodine and up to 30% of the need for selenium. The results indicate the possibility of increasing selenium accumulation during joint treatment of plants with iodide + selenate, as well as BISF, by 1.1–1.2 times.

### 3.6. Cost Analysis and Economic Feasibility of Biochar-Enriched Selenium and Iodine

The economic feasibility of biochar-enriched selenium and iodine as an alternative to traditional iodized sodium requires careful consideration of the costs associated with biochar production. The preparation of biochar involves several key cost factors, including the procurement of raw materials (plant biomass and chemicals such as KOH, selenium, and iodine), energy consumption during pyrolysis, and posttreatment processes. The pyrolysis process, which involves heating biochar samples to temperatures between 300°C and 850°C for several hours, is energy-intensive and contributes to higher operational costs than the simpler production process of iodized sodium. Additionally, biochar production requires more extensive posttreatment, such as washing and drying, to ensure its suitability for agricultural use (Table 3).

Despite these higher initial costs, biochar-enriched selenium and iodine offer significant long-term benefits that justify the investment. Biochar not only provides a stable and natural source of iodine and selenium in crops but also enhances soil health through improved carbon sequestration and nutrient retention. These environmental benefits, combined with potential long-term cost savings from increased crop yields and reduced fertilizer needs, make biochar-enriched selenium and iodine a cost-effective alternative in sustainable agriculture. While iodized sodium is a low-cost option for iodine supplementation, it lacks the broader agricultural and environmental benefits offered by biochar. Thus, the higher upfront costs of biochar are offset by its multifaceted advantages in both crop production and environmental remediation.

In summary, this approach offers several key advantages over traditional methods such as iodized salt, direct soil fertilization, and conventional biofortification techniques. Biochar acts as a stable and efficient carrier for selenium and iodine, ensuring a controlled and gradual release of these nutrients, thereby reducing the risk of leaching and nutrient loss that often occurs with direct soil fertilization. Additionally, biochar improves soil health by enhancing its physical properties, increasing water retention, and promoting carbon sequestration, benefits that traditional methods do not provide. Unlike iodized salt, which only addresses iodine deficiency, biochar-enriched crops deliver both selenium and iodine through a natural, plant-based source while simultaneously boosting crop yields and nutritional quality. This holistic approach not only addresses agricultural productivity and public health concerns but also integrates sustainable farming practices, making it a more effective and environmentally friendly alternative to conventional techniques.

## 4. Conclusions

This study presents a comprehensive investigation into the properties and applications of biochar derived from various biomass sources, focusing on their sorption capacities, structural characteristics, and potential agricultural benefits. The analysis revealed significant correlations between the iodine sorption capacity and the iodine number across different biochar samples, indicating that these parameters are crucial for determining the efficiency of biochar in adsorption applications. Among the biochar samples, AK biochar, which exhibited the best combination of specific surface area and iodine number, was selected for further enrichment treatments.

The comprehensive characterization of biochar through structural (SEM), elemental (EDAX), adsorption (nitrogen adsorption–desorption isotherms), and functional group (FTIR) analyses provided valuable insights into how the physical and chemical properties of biochar influence its effectiveness in both agricultural and environmental applications. These analyses supported the selection of AK biochar as the optimal choice for enrichment.

The study further explored the effects of various treatments—including selenium (Na_2_SeO_4_), iodine (KI), their combination (Na_2_SeO_4_ + KI), and BISF—on key biometric parameters of parsley. These treatments demonstrated a significant positive impact on both the growth and antioxidant properties of parsley, highlighting the potential of biochar as an effective carrier for nutrient delivery in agricultural systems.

While biochar production involves higher initial costs than traditional methods like iodized sodium, its long-term benefits—such as improved crop yields, enhanced nutritional quality, and environmental advantages—make it a cost-effective and sustainable alternative in agriculture. The broader implications of biochar use, including soil health improvement and carbon sequestration, further justify its adoption in sustainable farming practices.

## Figures and Tables

**Table 1:** Physicochemical characteristics of biochar samples.

Biochar type	Bulk density (kg/m^3^)	Average particle size (mm)	Specific surface area according to BET (m^2^/g)	Volume of micropores (cm^3^/g)	Mesopore volume (cm^3^/g)
RH	0.423 ± 0.029	0.80 ± 0.07	678.3 ± 65.4^a^	0.26 ± 0.03^a^	0.09 ± 0.01^a^
RS	0.396 ± 0.031^a∗^	0.35 ± 0.04	412.1 ± 41.7^b^	0.06 ± 0.01^b^	0.08 ± 0.01^a^
PS	0.482 ± 0.045^b^	2.50 ± 0.23	1206.4 ± 131.5^c^	0.46 ± 0.05^c^	0.16 ± 0.02^b^
WS	0.388 ± 0.038^a^	0.50 ± 0.05	369.4 ± 35.8^b^	0.07 ± 0.01^b^	0.07 ± 0.01^a^
AK	0.517 ± 0.047^b^	1.80 ± 0.20	983.8 ± 91.8^c^	0.57 ± 0.06^c^	0.17 ± 0.02^b^

*Note:*⁣^∗^Values with the same indices do not differ statistically at *p* < 0.05.

**Table 2:** The effect of selenium, iodine, and BASF on the accumulation of iodine and selenium compounds in plants.

Plant processing option	Iodine (mg/kg of d.w.)	Selenium (mcg/kg of d.w.)
Na_2_SeO_4_	—	2074.5 ± 191.8^a^
KI	7.1 ± 0.7^a∗^	—
Na_2_SeO_4_ + KI	17.1 ± 1.8^b^	2482.1 ± 201.3^b^
BISF	16.8 ± 1.7^b^	2358.4 ± 224.6^ab^

*Note:*⁣^∗^Values with the same indices do not differ statistically at *p* < 0.05.

**Table 3:** Cost comparison between biochar-enriched selenium/iodine and iodized sodium.

Cost factor	Biochar-enriched selenium/Iodine	Iodized sodium
Raw materials	Low-cost biomass (e.g., apricot kernel and sawdust) and chemicals (KOH, selenium, and iodine)	Low-cost sodium chloride and iodine fortification compounds [43]
Energy consumption	High (pyrolysis at 300°C–850°C for 3 h)	Low (simple mixing and drying processes) [44]
Posttreatment	Costs for washing with distilled water and drying	Minimal postprocessing required
Nutritional benefits	Provides a stable and natural source of iodine and selenium in crops (e.g., parsley)	Provides iodine primarily through salt consumption
Environmental benefits	Improves soil health, carbon sequestration, and nutrient retention in soil	Limited to iodine fortification, no environmental benefits
Long-term cost savings	Potential savings due to improved crop yields and reduced fertilizer needs	None, limited to iodine supplementation
Overall cost	Higher initial cost, but with additional long-term benefits in agriculture and sustainability	Lower cost, but limited to short-term iodine supplementation benefits

## Data Availability

The data used to support the findings of this study are available from the corresponding author upon reasonable request.

## Supporting Information

Supporting Information Graphical scheme of the biochar-based granular iodine–selenium complex fertilizer preparation process and its application for biofortification of parsley.
