# Supplementary figures and images for: Preparation of Granular Organic Iodine and Selenium Complex Fertilizer Based on Biochar for Biofortification of Parsley
Source: Scientifica (Cairo). 2024 Oct 21;2024:6601899. doi: 10.1155/2024/6601899 (PMC11519072; doi:10.1155/2024/6601899)

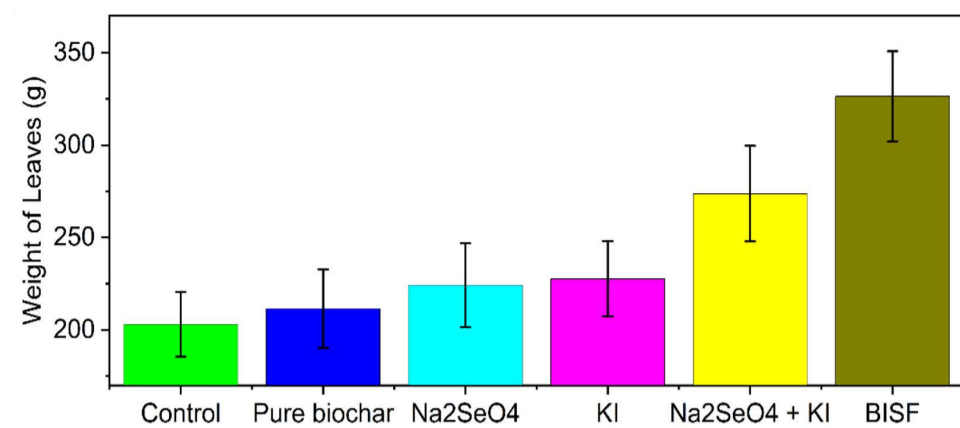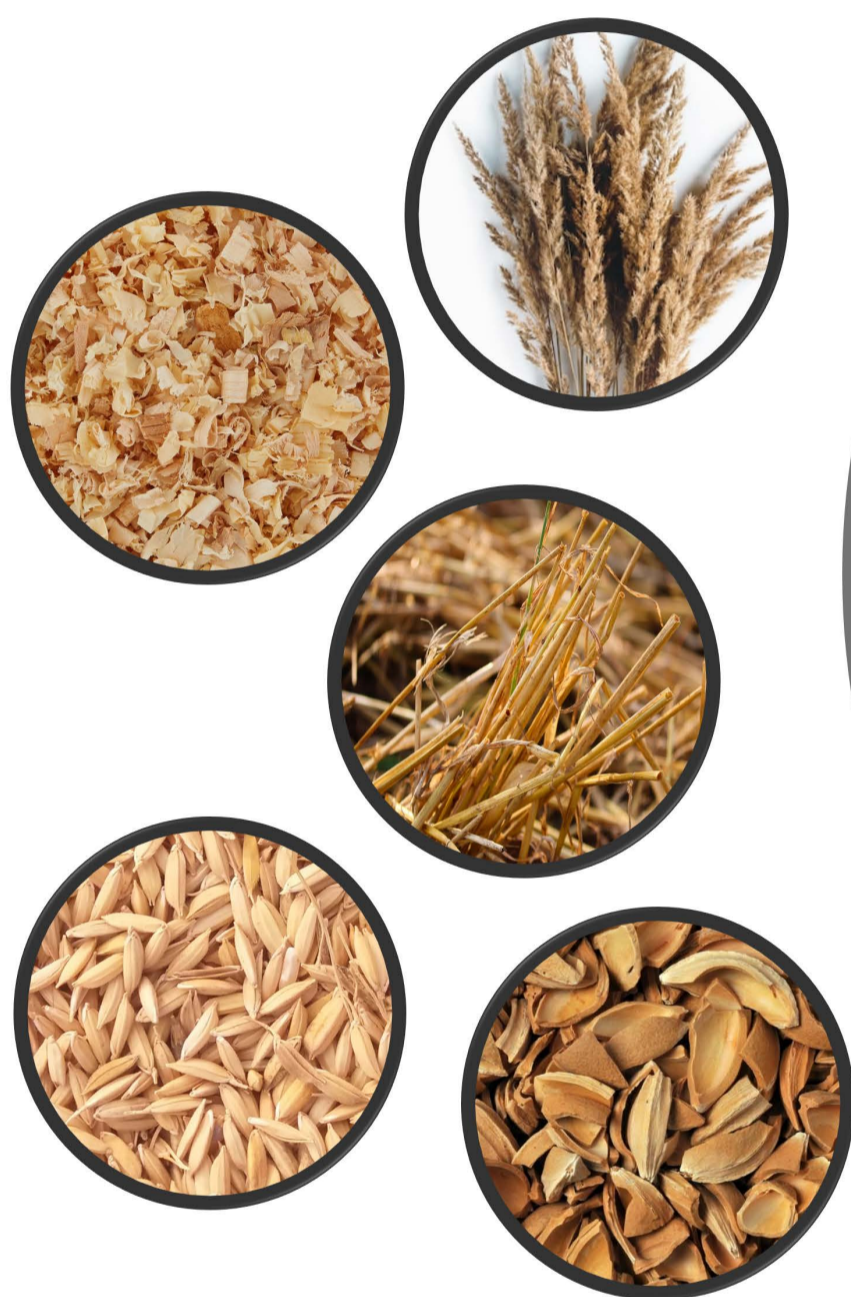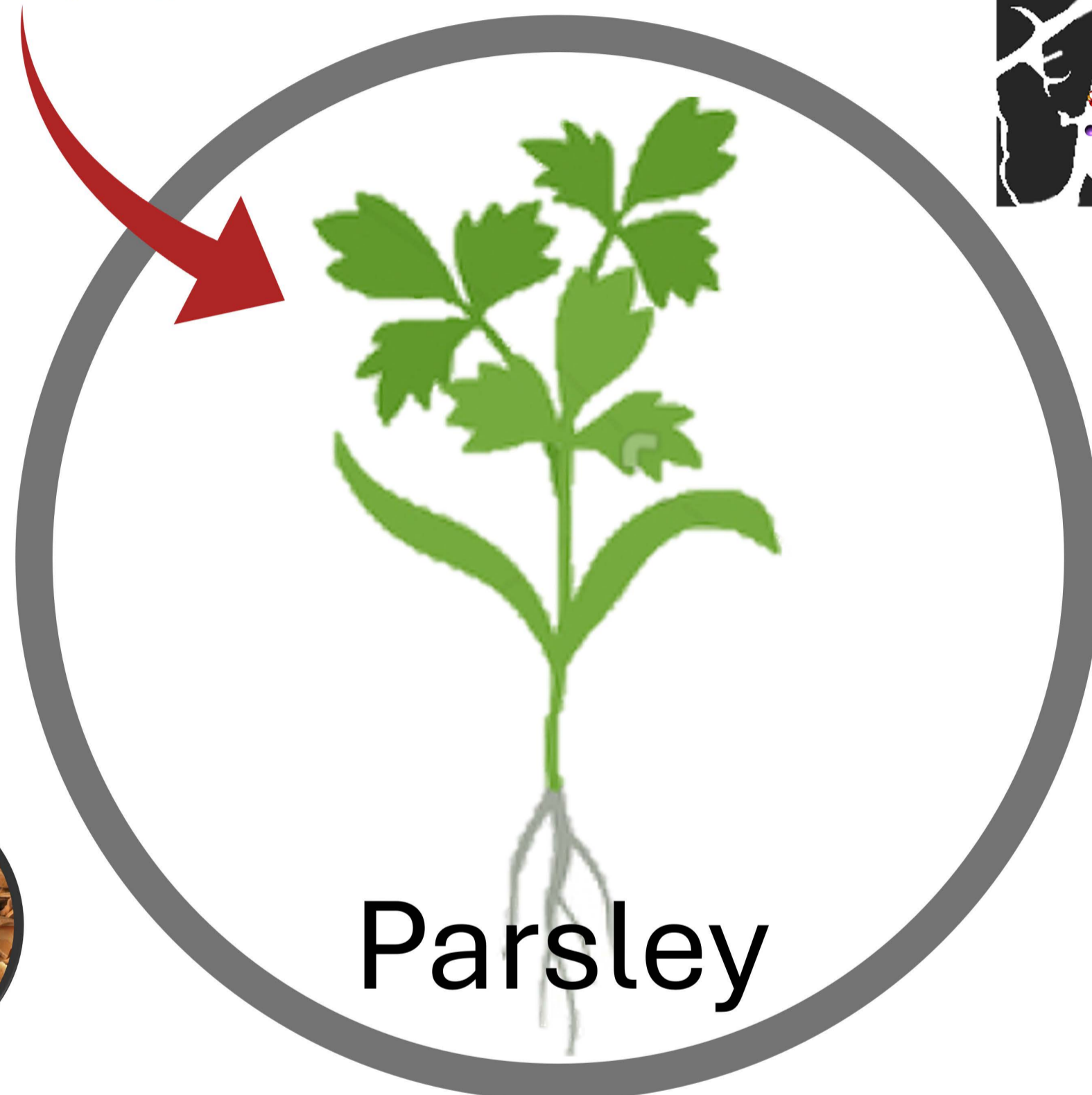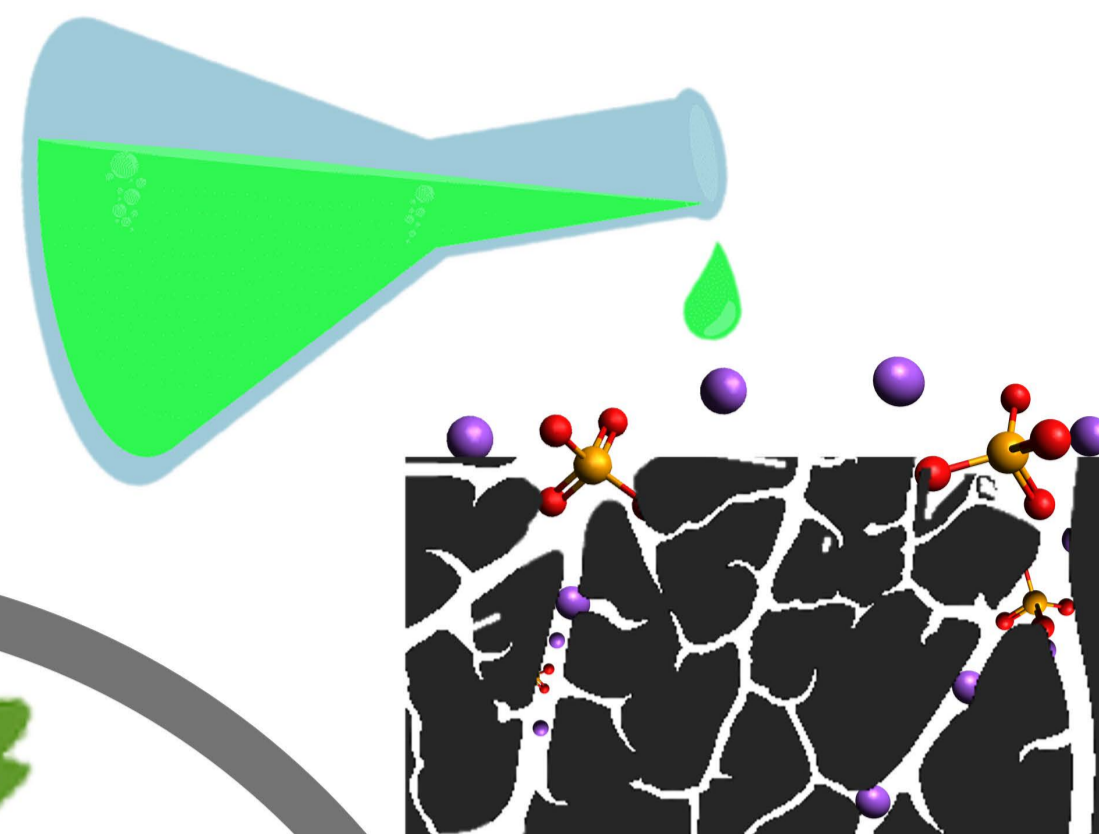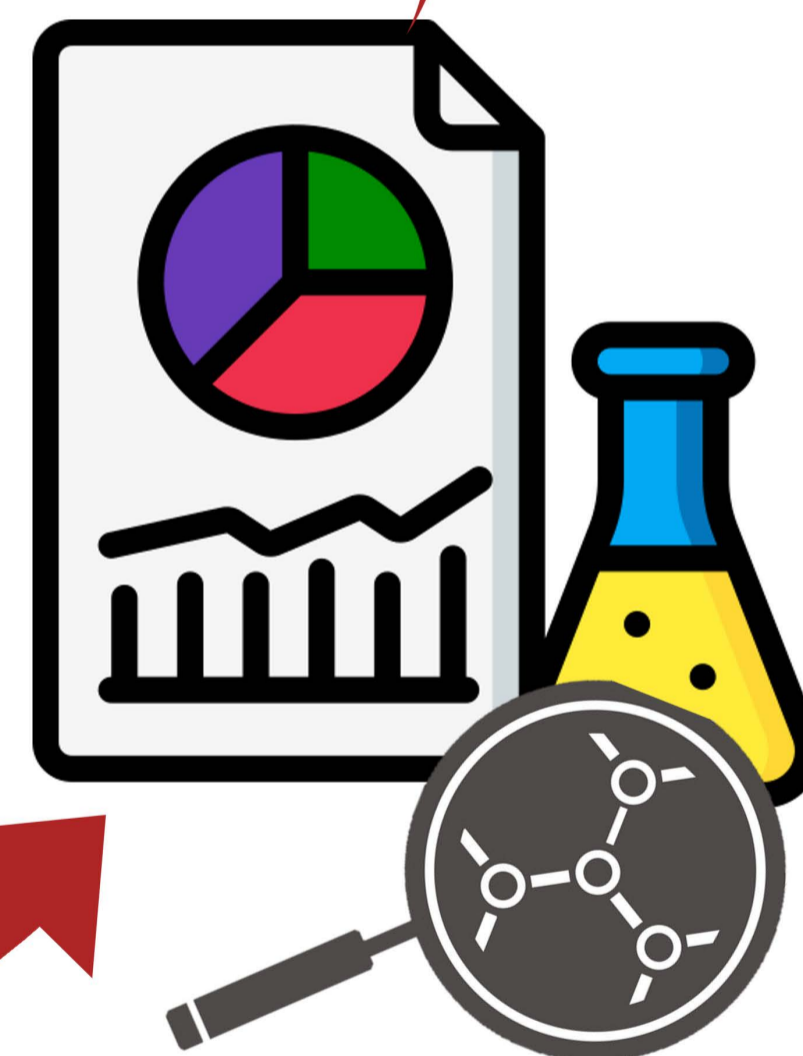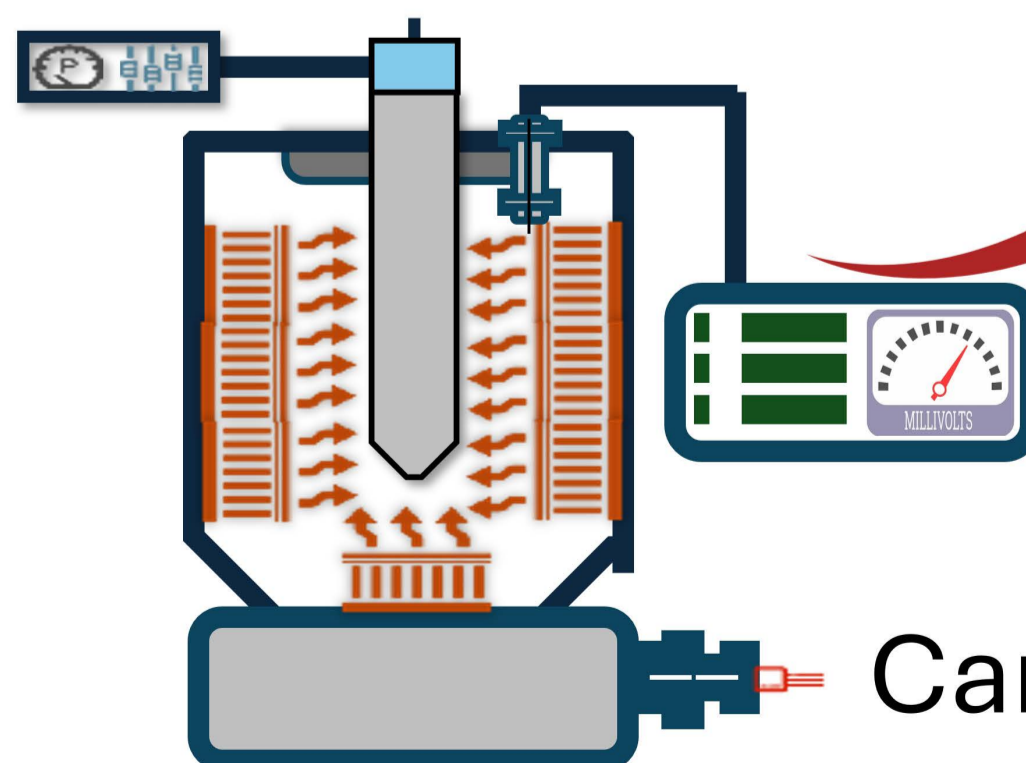

Carbonization

Supplement: Supporting Information — Graphical scheme of the biochar-based granular iodine–selenium complex fertilizer preparation process and its application for biofortification of parsley. [file 6601899.f1.pdf]
